# Clinical characteristics and population‐based attack rates of respiratory syncytial virus versus influenza hospitalizations among adults—An observational study

**DOI:** 10.1111/irv.12914

**Published:** 2021-10-03

**Authors:** Raija Auvinen, Ritva Syrjänen, Jukka Ollgren, Hanna Nohynek, Kirsi Skogberg

**Affiliations:** ^1^ Inflammation Center University of Helsinki and Helsinki University Hospital Helsinki Finland; ^2^ Infectious Disease Control and Vaccinations Unit, Department of Health Security Finnish Institute for Health and Welfare Helsinki Finland; ^3^ Internal Medicine and Rehabilitation University of Helsinki and Helsinki University Hospital Helsinki Finland; ^4^ Population Health Unit, Department of Public Health and Welfare Finnish Institute for Health and Welfare Helsinki Finland

**Keywords:** adult, hospitalization, incidence, influenza, respiratory syncytial viruses

## Abstract

**Background:**

The clinical significance of respiratory syncytial virus (RSV) among adults remains underinvestigated. We compared the characteristics and population‐based attack rates of RSV and influenza hospitalizations.

**Methods:**

During 2018–2020, we recruited hospitalized adults with respiratory infection to our prospective substudy at a tertiary care hospital in Finland and compared the characteristics of RSV and influenza patients. In our retrospective substudy, we calculated the attack rates of all RSV and influenza hospitalizations among adults in the same geographic area during 2016–2020.

**Results:**

Of the 537 prospective substudy patients, 31 (6%) had RSV, and 106 (20%) had influenza. Duration of hospitalization, need for intensive care or outcome did not differ significantly between RSV and influenza patients. RSV was more often missed or its diagnosis omitted from medical record (13% vs 1% *p* = 0.016 and 48% vs 15%, *p* > 0.001). In the retrospective substudy, the mean attack rates of RSV, influenza A, and influenza B hospitalizations rose with age from 4.1 (range by season 1.9–5.9), 15.4 (12.3–23.3), and 4.7 (0.5–16.2) per 100,000 persons among 18‐ to 64‐year‐olds to 58.3 (19.3–117.6), 204.1 (31.0–345.0), and 60.4 (0.0–231.0) per 100,000 persons among 65+‐year‐olds and varied considerably between seasons.

**Discussion:**

While the attack rates of influenza hospitalizations were higher compared with RSV, RSV and influenza hospitalizations were similar in severity. Missing or underreporting of RSV infections may lead to underestimating its disease burden. Both RSV and influenza caused a substantial amount of hospitalizations among the elderly, stressing the need for more effective interventions.

## 1 INTRODUCTION

While influenza is a widely acknowledged global health threat, the burden of respiratory syncytial virus (RSV) remains less well‐charted among adults. Previously recognized as an important pathogen among children, RSV is now known to cause notable morbidity and mortality among adults, especially among immunocompromised individuals, those with cardiopulmonary diseases, and 65+‐year‐old elderly.^1^, ^2^, ^3^, ^4^, ^5^, ^6^, ^7^, ^8^ Studies suggest that RSV infection develops yearly in 3–7% of healthy elderly adults and 4–10% of high‐risk adults with chronic heart or lung disease, whereas seasonal influenza infects about 10% of adults, half of whom have symptomatic infection.^6^, ^9^, ^10^ RSV has repeatedly been among the top five causative agents of viral respiratory infections in the elderly along with influenza.^7^, ^11^, ^12^

Influenza offers a practical point of comparison to RSV as they circulate simultaneously, and the same clinical criteria and sample can be used for testing. Both RSV and influenza epidemics occur between Weeks 41 and 20 in the northern hemisphere.^13^, ^14^ In Finland, RSV epidemics used to follow a pattern of a minor peak in April followed by a major peak in December every 2 years; however, since 2008, RSV epidemics occur every winter peaking between February and May but still exhibit a biennial pattern of major and minor epidemics.^15^, ^16^ Influenza epidemics vary considerably between seasons depending on the circulating virus type and preexisting immunity of the population. Previously, insensitive or slow diagnostic methods such as viral culture and serology were used to detect RSV in clinical studies, while tests suitable for clinical use were lacking.^5^, ^17^ Recently, molecular polymerase chain reaction (PCR)‐based diagnostic methods have enabled the fast and accurate diagnostics of RSV alongside influenza testing.^17^, ^18^, ^19^

Although RSV hospitalizations have been studied among children, elderly, and high‐risk adults, few recent prospective studies have investigated all 18+ hospitalized adults.^20^, ^21^, ^22^, ^23^ A more complete picture of the RSV disease burden and potential vaccine target groups among adults is required as RSV vaccines likely become available soon.^24^, ^25^

We compared the clinical characteristics of adults hospitalized with RSV or influenza and calculated the population‐based seasonal attack rates of RSV and influenza hospitalizations among adults to estimate their respective disease burdens in different age groups.

## 2 METHODS

### 2.1 Study design

Our study included two substudies: a prospective hospital‐based substudy and a retrospective register‐based cohort substudy of 18+ adults hospitalized with laboratory‐confirmed RSV or influenza. Prospective substudy data were used to compare the clinical characteristics of RSV and influenza and to assess the proportion of all severe acute respiratory infection (SARI) hospitalizations attributable to these viruses. Retrospective register data were used to compare the age and sex distributions of patients hospitalized with RSV or influenza and to calculate population‐based seasonal attack rates of RSV and influenza hospitalizations.

### 2.2 Study setting and population

Jorvi Hospital is a tertiary care hospital belonging to HUS Helsinki University Hospital (HUS) and serving a catchment area consisting of the populations of the municipalities of Espoo, Kirkkonummi, and Kauniainen. All residents of these three municipalities requiring hospital care are referred to Jorvi Emergency Clinic (JEC), from where patients are admitted to Jorvi Hospital or adjacent Espoo Hospital for secondary care. Exceptions occur when patients requiring specific neurologic or surgical treatments are directly referred to other HUS hospitals. Additionally, patients with acute hematologic malignancy, recent solid organ transplant, or HIV infection are not treated at Jorvi Hospital. During 2016–2020, 248,000–262,000 adults resided in the JEC catchment area (study area) and formed the background population of both substudies.

#### 2.2.1 Prospective hospital‐based substudy

We conducted a prospective observational substudy on adult patients hospitalized with SARI requiring tertiary care at Jorvi Hospital, Espoo, Finland, during two epidemic seasons 2018–2020. Ethics approval was granted by the Local Ethics Committee of HUS, and study permits were obtained from HUS, the Finnish Institute for Health and Welfare (THL), and municipalities involved. The study was a part of an international DRIVE collaboration (www.drive-eu.org) and followed its test‐negative design (TND) influenza vaccine effectiveness (IVE) core protocol.^26^ Although the main focus was on estimating IVE, study objectives included assessing the disease burdens of influenza and RSV. All wards treating adult patients with respiratory infection were included as study wards. Patients were recruited from November 23, 2018, to April 30, 2019, and from December 2, 2019, to June 12, 2020. A comparison of the COVID‐19 and influenza patients recruited to the substudy in 2019–2020 has been published, and COVID‐19 patients were excluded from this study.^27^

All hospitalized SARI patients were screened, and eligible patients were recruited by study nurses. Community‐dwelling 18+ adults living in the study area were included. SARI patient was defined as having at least one respiratory (cough, dyspnea, sore throat) and one systemic symptom (fever or feverishness, headache, myalgia, malaise, deterioration of general condition) with symptoms having started at most 7 days before sampling and before or within 48 h from admission. Patients previously hospitalized less than 48 h before the onset of the first SARI symptom were excluded. Informed consent from either patient or (in the case of severely ill patients) from their next of kin was required. Respiratory samples were obtained for influenza and RSV testing. Coinfections of RSV and influenza were excluded from analyses.

Background data were gathered from patient interviews and electronic medical records, National Vaccination Register (NVR), and Care Register for Health Care (HILMO) by study nurses and physicians. Symptoms experienced by the time of the interview were recorded. Follow‐up lasted 3 months from admission. Collected data included comorbidities, smoking, influenza vaccinations, previous hospitalizations during the past 12 months, intensive care unit (ICU), and close observation admissions, outcome, and readmissions. Previous overnight hospitalizations were verified from the HILMO register. The severity of chronic conditions was assessed using the McCabe score (1 = nonfatal; 2 = fatal in 1–4 years; 3 = fatal within a year).^28^

#### 2.2.2 Retrospective register‐based substudy

To calculate acute hospitalizations with laboratory‐confirmed RSV or influenza occurring in our background population, we obtained a record of all adult patients who tested positive for RSV or influenza at JEC during four epidemic seasons 2016–2020. As the study was a register‐based study and personal medical records were not accessed, no ethics approval was required. The study protocol was institutionally approved.

The record, obtained from HUS Information Management and Technology Unit, contained pseudonymized data on all adult patients whose positive RSV or influenza test was taken at JEC between November 1 and May 31 during years 2016–2020. We limited the study period to the epidemic season of November–May because, virtually, all cases of both RSV and influenza occur during this period in Finland.^14^ The record included information on patients' age, sex, home municipality, the type of positive laboratory test (virus type, antigen detection or PCR), and whether the patients were hospitalized or discharged from JEC. Patients hospitalized at a secondary or tertiary care hospital were included in further analyses. Patients residing outside the study area or under 18 years old were excluded from analyses. Patients were included in the record several times during one epidemic season if they were positive for different viruses on separate occasions. Coinfections of RSV and influenza or different influenza types were excluded from analyses (Table S1).

Demographics of residents living in the JEC catchment area on December 31, 2016, 2017, 2018, and 2019 were obtained from Statistics Finland's PxWeb databases.^29^ Detections of RSV and influenza in the study area were obtained from the National Infectious Diseases Register (NIDR) containing all positive laboratory findings of these pathogens electronically reported by clinical microbiology laboratories operating in the area.

### 2.3 Laboratory testing

All patients included in the prospective substudy were tested for influenza and RSV by real‐time reverse transcription PCR (RT‐PCR) either at Helsinki University Hospital laboratory (HUSLAB) or at THL Expert Microbiology Unit, where all influenza‐positive samples were subtyped. Diagnostic respiratory samples were usually taken from the nasopharynx by hospital staff. If no clinical samples were taken, study samples were taken from both nostrils and throat by the study nurses. At HUSLAB, influenza and RSV testing was performed using Xpert®Xpress Flu/RSV assay (Cepheid, Sunnyvale, CA, USA). At THL, an in‐house PCR was used.^30^

Laboratory testing of retrospective cohort patients examined at JEC was clinician‐driven and done at HUSLAB according to local guidelines stating that during epidemic seasons, all patients hospitalized with suspected respiratory infection should be tested for influenza. During epidemic seasons 2016–2019, respiratory samples were first tested with rapid influenza antigen detection test (RIADT, Actim® Influenza A&B, Medix Biochemica), and negative results were always confirmed by the aforementioned RT‐PCR test including an RSV test. Since autumn 2019, the use of RIADT was discontinued due to low sensitivity observed during previous years, and only direct RT‐PCR testing for both influenza (A and B) and RSV was conducted.

In March 2020, the COVID‐19 pandemic spread to the study area after which all hospitalized SARI patients were tested for SARS‐CoV‐2 while diagnostic testing for influenza or RSV was not always done. However, all patients included in the prospective substudy since March 2020 were tested for influenza and RSV whenever a respiratory sample was available (66/94, 70%).

### 2.4 Data analysis

Using prospective substudy data, we compared the clinical characteristics, symptoms, and outcomes of RSV and influenza patients. Categorical variables were compared using Fisher's exact test and continuous variables using Mann–Whitney's *U* test. Data analysis was performed using SPSS version 26.0 (IBM SPSS statistics®).

With the use of retrospective register data, we calculated seasonal attack rates (or cumulative incidences) of hospitalizations by dividing the number of hospitalized patients positive for RSV or influenza in a certain age group during an epidemic season by the base population in that age group on December 31 during that season. Mean attack rates over four seasons were calculated from seasonal attack rates.

## 3 RESULTS

### 3.1 Results of the prospective substudy

Of the 537 SARI patients included in the prospective substudy, 31 (6%) had RSV, 106 (20%) had influenza, and one had a coinfection of RSV and influenza A. RSV cases were less often detected from clinical samples than influenza cases (27/31, 87% vs. 105/106, 99%, *p* < 0.016), and the rest were detected from study samples. During season 2018–2019, RSV was detected in 11/293 (3.8%) and influenza in 73/293 (24.9%) of SARI patients. During season 2019–2020, RSV was detected in 20/244 (8.2%) and influenza in 33/244 (13.5%) of SARI patients. Influenza subtypes were the following: 29 (40%) were A(H1pdm09) and 44 (60%) A(H3N2) in 2018–2019, and 29 (88%) were A(H1N1)pdm09, 2 (6%) A(H3N2), and 2 (6%) B Victoria in 2019–2020.

In the prospective substudy, RSV patients were older than influenza patients with a median age of 73 years (interquartile range [IQR] 64–82, range 47–89) versus 67 years (IQR 48–75, range 18–92, *p* = 0.014, Table 1). When compared by influenza subtype, A(H1N1)pdm09 patients were younger than A(H3N2) patients (median age 62.5 vs. 71.0, *p* = 0.014, data not shown). The majority (18, 58%) of RSV and nearly half (51, 48%) of influenza patients were women.

**TABLE 1:** Clinical characteristics of RSV and influenza patients in the prospective substudy in 2018–2020

Characteristics	RSV patients		Influenza patients		*p* value
	*N*	%	*N*	%	
Total	31	100	106	100	
Sex					
Female	18	58	51	48	0.415
Male	13	42	55	52	
Age (in years)					
Median (IQR) [range]	73 (64–82) [47–89]	N/A	67 (48–75) [18–92]	N/A	0.014
18–64	9	29	47	44	0.149
65 or over	22	71	59	56	
Comorbidities					
Anemia	2	6	11/105	10	0.732
Cancer	11	35	20	19	0.085
Cardiovascular disease	12	39	36	34	0.671
Diabetes	12	39	29	27	0.266
Hypertension	22	71	60	57	0.211
Immunosuppression	7	23	12	11	0.139
Kidney disease	8	26	9	8	**0.025**
Liver cirrhosis or failure	1	3	4	4	1.000
Neurological disease	2	6	12	11	0.736
Obesity (BMI ≥ 30)	11	35	36	34	1.000
Pulmonary disease (incl. sleep apnea)	16	52	49/104	47	0.687
Rheumatic disease	4	13	8/105	8	0.469
Stroke	5	16	10	9	0.329
No underlying conditions	1	3	12/105	11	0.297
McCabe score					
1	18	58	79	75	0.114
2 or 3	13	42	27	25	
Smoking					
Never	9	29	49	46	0.161
Ex smoker	16	52	32	30	
Current smoker	6	19	25	24	
Hospitalized in last 12 months					
Yes	10	32	29	27	0.653
Influenza vaccination (current season)					
Yes, season 2018–2019	6	55	42	58	1.000
Yes, season 2019–2020	12	60	15	45	0.398
Symptom onset to hospitalization					
Symptom day at hospitalization, median (IQR) [range]	4 (3–5) [1–8]	N/A	3 (2–4) [1–8]	N/A	**0.006**
0–3 days	20	65	85	80	0.091
4–7 days	11	35	21	20	
Reported symptoms					
Fever ≥ 38	23	74	96	91	**0.031**
Feverishness (37.0–37.9)	6	19	10	9	0.199
Malaise	2	6	2	2	0.220
Headache	13	42	58/104	56	0.220
Myalgia	13	42	51/104	49	0.543
Deterioration of general condition	27	87	100	94	0.233
Cough	31	100	97	92	0.209
Sore throat	17	55	48/105	46	0.417
Dyspnea	22	71	80	75	0.643
Sudden onset	7	23	62/102	61	**<0.001**
Outcomes during hospitalization					
Duration of hospitalization at HUS, median (IQR) [range]	4 (3–6) [1–22]	N/A	3 (2–6) [1–46]	N/A	0.510
Admission to close observation	1	3	8	8	0.684
Admission to ICU	0	0	6	6	0.337
Death	0	0	0	0	N/A
Clinical recognition					
Only detected from study sample^a^	4	13	1	1	**0.016**
Virus specific ICD‐10 code in medical record^b^	16	52	90	85	**<0.001**
Outcome at 30 days from admission					
Discharged	31	100	100	94	0.745
Still hospitalized at tertiary care hospital	0	0	2	2	
Hospitalized at secondary care hospital	0	0	4	4	
Dead	0	0	0	0	
Outcome at 3 months from admission					
Readmitted at least once	4	13	20	19	0.594
Dead	2	6	1	1	0.128

*Note*: Data are presented as No. and % or as medians with interquartile ranges (IQRs). If data are missing, the proportion of patients with available information is marked as the denominator.

Abbreviations: BMI, body mass index; HUS, HUS Helsinki University Hospital; ICU, intensive care unit; RSV, respiratory syncytial virus.

^a^ Sample obtained by the study nurse, when no diagnostic sample was taken for clinical use.

^b^ J09–J11 for influenza, J12.1 or J20.5 for RSV.

All except one (30, 97%) of RSV patients had at least one comorbidity compared with 93/105 (89%) of influenza patients (Table 1). Hypertension, pulmonary, and cardiovascular diseases were the most common comorbidities in both RSV and influenza patients. Data suggested that kidney diseases were more common among RSV than influenza patients (8, 26% vs. 9, 8%, *p* = 0.025), and RSV patients were slightly more likely to have cancer (11, 35% vs. 20, 19%, *p* = 0.085). McCabe score was 2 or 3 indicating the presence of serious comorbidities in 42% of RSV and 25% of influenza patients (*p* = 0.114).

Almost a third of both RSV and influenza patients had been hospitalized in the previous 12 months (10, 32% vs. 29, 27%). Smoking was as common in both groups with six (19%) of RSV and 25 (24%) of influenza patients being current smokers. Uptake of seasonal influenza vaccine (14+ days before hospitalization) did not differ significantly between RSV and influenza patients (Table 1).

Sudden onset of symptoms was less common among RSV than influenza patients (23% vs. 61%, *p* < 0.001, Table 1). Only 74% of the RSV patients had fever ≥38°C compared with 91% of influenza patients (*p* = 0.031). Cough was the most common symptom among both RSV and influenza patients followed by deterioration of general condition and dyspnea.

RSV patients were hospitalized on average on Day 4 of symptoms (IQR 3–5) compared with Day 3 (IQR 2–4) for influenza patients (*p* = 0.006, Table 1). The durations of their hospitalizations were similar (median 4 days, IQR 3–6 vs. 3, 2–6, *p* = 0.510). Of the RSV and influenza patients, 1 (3%) and 8 (8%) were admitted to close observation, and none of RSV and 6 (6%) of influenza patients needed ICU care. None of the patients died in the hospital.

By Day 30 from admission, all RSV and 100 (94%) of influenza patients had been discharged (Table 1). The virus‐specific ICD‐10 diagnosis (J09‐J11 for influenza, J12.1 or J20.5 for RSV) was less often included in the medical record of RSV than influenza patients (48% vs. 15%, p < 0.001). By 3 months from admission, 4 (13%) of RSV and 20 (19%) of influenza patients had been readmitted to the hospital, and 2 (6%) and 1 (1%) had died.

### 3.2 Results of the retrospective register‐based substudy

**FIGURE 1:**
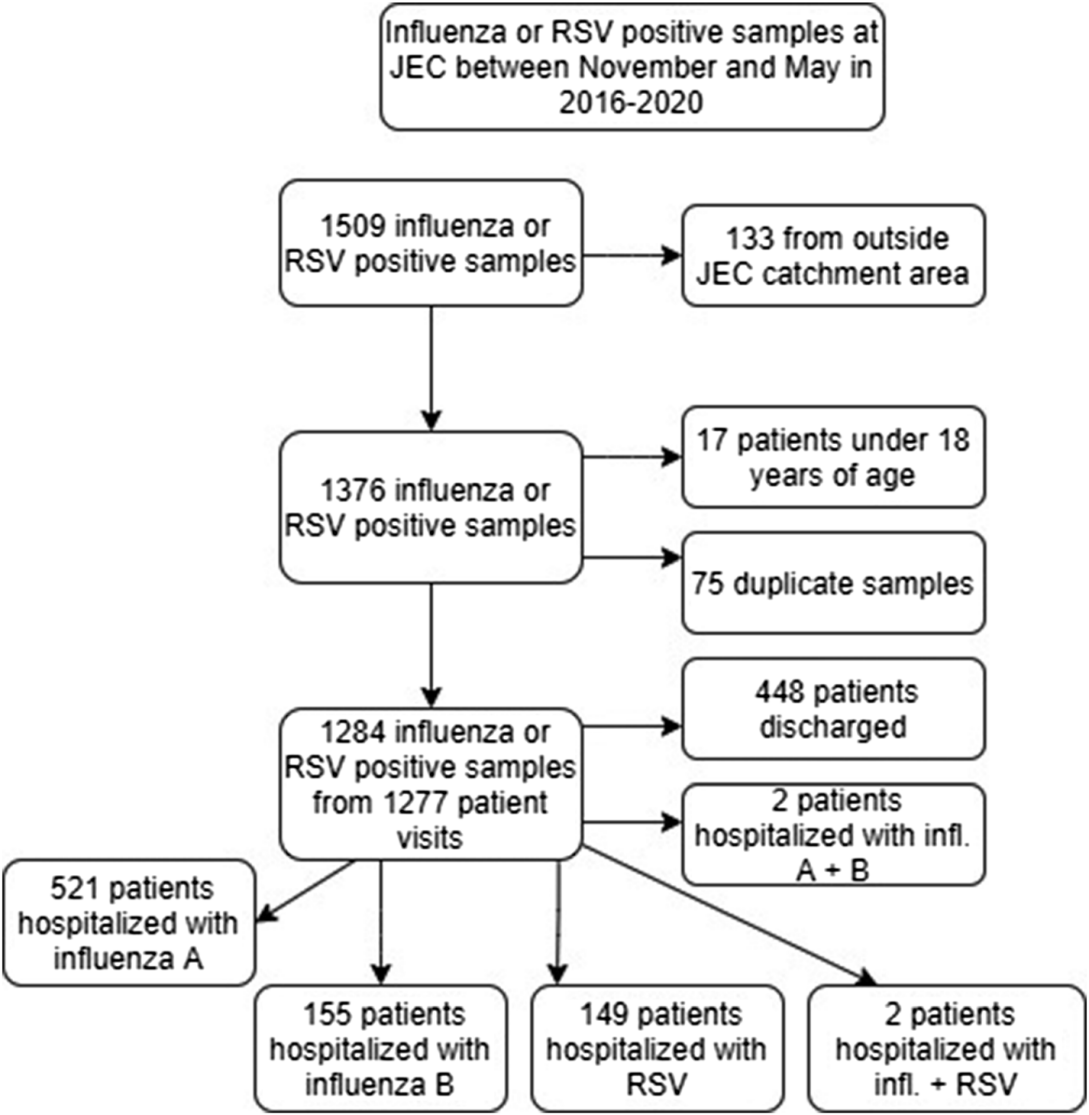
Flowchart of included and excluded patients of the retrospective substudy

During epidemic seasons in 2016–2020, 1509 RSV or influenza‐positive samples were taken at JEC from adult patients, and altogether, 825 patients living in the study area were hospitalized with RSV or influenza (Figure 1). Majority (521, 62.8%) were hospitalized with influenza A and similar numbers with influenza B (155.18.7%) and RSV (149.18.0%). Additionally, two patients had influenza A + B, one influenza A + RSV, and one influenza B + RSV. Of these 825 patients, 208 patients (15 RSV, 193 influenza) were hospitalized in 2016–2017, 350 (69 RSV, 281 influenza) in 2017–2018, 184 (26 RSV, 158 influenza) in 2018–2019, and only 83 (39 RSV, 44 influenza) in 2019–2020 (Table 2).

**TABLE 2:** Number, age, and sex distribution of retrospective cohort patients hospitalized with RSV, influenza A, or influenza B during epidemic seasons 2016–2020

Characteristics	Total		RSV		Influenza A		Influenza B		Influenza total		*p* value
	*N*	%	*N*	%	*N*	%	*N*	%	*N*	%	
Total	825	100	149	100	521	100	155	100	676	100	N/A
Epidemic season											
2016–2017	208	25.2	15	10.1	186	35.7	7	4.5	193	28.6	N/A
2017–2018	350	42.4	69	46.3	136	26.1	145	93.5	281	41.6	
2018–2019	184	22.3	26	17.4	157	30.1	1	0.6	158	23.4	
2019–2020	83	10.1	39	26.2	42	8.1	2	1.3	44	6.5	
Sex											
Women	464	56.2	90	60.4	277	53.2	97	62.6	374	55.3	0.062
Men	361	43.8	59	39.6	244	46.8	58	37.4	302	44.7	
Age											
Median (IQR) [range]	76 (65.84) [18–100]		77 (67.86) [22–97]		76 (65.84) [18–100]		75 (65.83) [19–100]				0.456
18–64 years	199	24.1	34	22.8	127	24.4	38	24.5	165	24.4	0.772
65–84 years	427	51.8	73	49.0	271	52.0	83	53.5	354	52.4	
85+ years	199	24.1	42	28.2	123	23.6	34	21.9	157	23.2	

Abbreviations: IQR, interquartile range; RSV, respiratory syncytial virus.

Age and sex distributions of the patients hospitalized with RSV or influenza A or B did not differ significantly over the whole study period (Table 2). The median ages of RSV, influenza A, and influenza B patients were 77, 76, and 75 years, respectively. Elderly patients represented 77.2% of RSV, 76.6% of influenza A, and 75.4% of influenza B patients. However, when the analysis was limited to 2018–2020 as in our prospective substudy, RSV patients were significantly older than influenza patients (median age 77.0 vs. 71.5, *p* = 0.005). Age and sex distribution of hospitalized patients by epidemic season and virus type are shown in Table S1.

**FIGURE 2:**
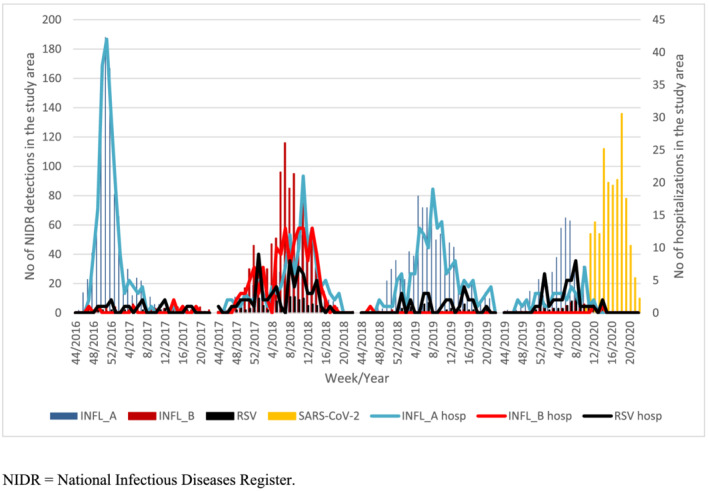
Weekly respiratory syncytial virus (RSV), influenza, and SARS‐CoV‐2 detections in National Infectious Diseases Register (NIDR) (bars) and RSV and influenza hospitalizations (lines) among adults in the study area

**FIGURE 3:**
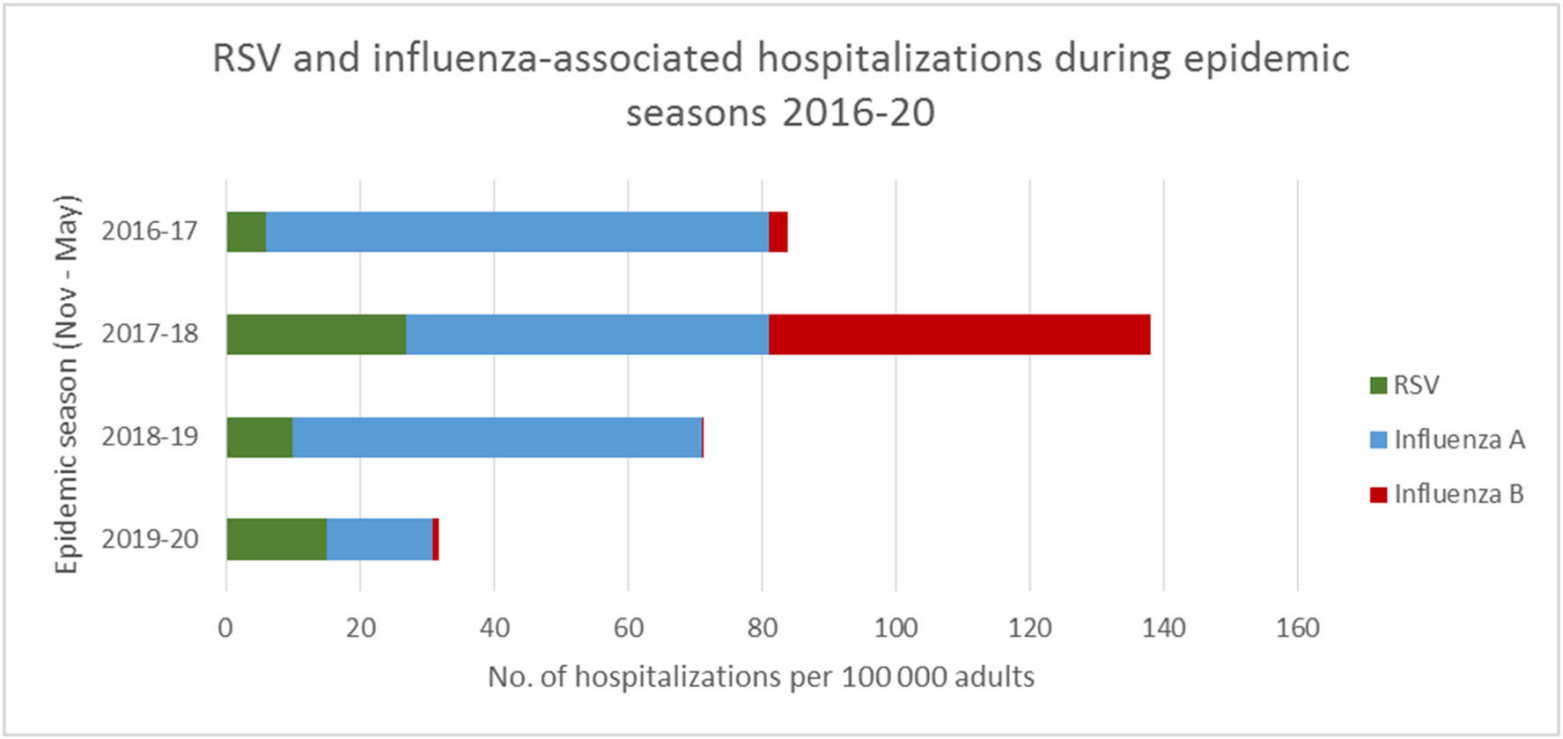
Respiratory syncytial virus (RSV) and influenza hospitalizations per 100,000 adults during epidemic seasons 2016–2020 in the study area

There was a considerable season‐to‐season variation in both RSV and especially influenza hospitalizations (Figures 2 and 3). RSV hospitalizations ranged from 15 to 69 hospitalizations per epidemic season and exhibited a biannual pattern with the highest amounts of hospitalizations in 2017–2018 and 2019–2020 (Table 2). Influenza hospitalizations ranged from 44 in 2019–2020 to 281 in 2017–2018 with the highest number of influenza A hospitalizations (186) observed in 2016–2017. Variability was most obvious in the case of influenza B, which caused few (7) hospitalizations in 2016–2017, more hospitalizations than influenza A (145) in 2017–2018, and virtually no hospitalizations (1 and 2, respectively) in 2018–2019 and 2019–2020. While the dominating influenza subtype changed from season to season, vaccination coverage among the elderly population in the study area as obtained from NVR was relatively constant at 54.6%–57.9% (Table 3). During season 2019–2020, both RSV and influenza detections and hospitalizations rapidly decreased after nationwide lockdown due to the COVID‐19 pandemic on March 17, 2020 (Week 12) (Figure 2).

The mean attack rates of acute RSV, influenza A, and influenza B hospitalizations per 100,000 adults over four epidemic seasons were 14.6 (range 6.0–27.4), 51.6 (16.0–75.0), and 15.4 (0.4–57.5), respectively (Table 3). The total seasonal burden of influenza hospitalizations was highest (111.4 per 100,000 adults) in 2017–2018 due to circulation of both A and B influenza during the season. Together, RSV and influenza hospitalizations ranged from 31.8 per 100,000 adults in 2019–2020 to 138.7 per 100,000 in 2017–2018 (Figure 3).

Population‐based seasonal attack rates of acute RSV and influenza hospitalizations per 100,000 adults by age groups and epidemic seasons are depicted in Table 3. Over four epidemic seasons, mean attack rates of influenza were four to fivefold compared with RSV. Attack rates consistently increased with age for both RSV and influenza. Among 18‐ to 64‐year‐olds, the mean attack rates of RSV, influenza A, and influenza B hospitalizations were 4.1 (range 1.9–5.9), 15.4 (12.3–23.3), and 4.7 (0.5–16.2) per 100,000 persons. Among the 65+ elderly, the mean attack rates of RSV, influenza A, and influenza B hospitalizations were 58.3 (range 19.3–117.6), 204.1 (31.0–345.0), and 60.4 (0–231.0) per 100,000 persons. The highest mean attack rates occurred among 85+ persons, who had 219.8 (range 112.3–386.5) of RSV, 667.5 (58.6–1212.9) of influenza A, and 183.0 (0–687.1) of influenza B hospitalizations per 100,000 persons.

**TABLE 3:** Seasonal attack rates of RSV and influenza hospitalizations per 100,000 persons by epidemic season and age group with 95% confidence intervals, mean attack rates over the whole study period, and seasonal circulating virus types and vaccination coverages

Age group	Virus type	2016–2017	2017–2018	2018–2019	2019–2020	Mean, 2016–2020
18+ adults	RSV	6.0 (3.4, 10.0)	27.4 (21.3, 34.6)	10.2 (6.6, 14.9)	14.9 (10.6, 20.3)	14.6
	Infl. A	75.0 (64.6, 86.5)	53.9 (45.2, 63.8)	61.3 (52.1, 71.6)	16.0 (11.5, 21.6)	51.6
	Infl. B	2.8 (1.1, 5.8)	57.5 (48.5, 67.6)	0.4 (0.0, 2.2)	0.8 (0.1, 2.8)	15.4
18‐ to 64‐year‐olds	RSV	3.0 (1.1, 6.5)	5.9 (3.0, 10.3)	1.9 (0.5, 5.0)	5.7 (2.9, 9.9)	4.1
	Infl. A	12.4 (8.0, 18.3)	13.7 (9.1, 19.9)	23.3 (17.2, 30.9)	12.3 (8.1, 18.1)	15.4
	Infl. B	1.0 (0.1, 3.6)	16.2 (11.2, 22.7)	0.5 (0.0, 2.7)	0.9 (0.1, 3.4)	4.7
65+ elderly	RSV	19.3 (8.8, 36.6)	117.6 (89.1, 152.3)	43.9 (27.5, 66.5)	52.3 (34.5, 76.1)	58.3
	Infl. A	345.0 (293.9, 402.5)	222.7 (182.8, 268.9)	217.7 (178.8, 262.6)	31.0 (17.7, 50.4)	204.1
	Infl. B	10.7 (3.5, 25.0)	231.0 (190.2, 277.9)	0.0 (0.0, 7.4)	0.0 (0.0, 7.2)	60.4
65‐ to 84‐year‐olds	RSV	9.5 (2.6, 24.3)	89.0 (63.3, 121.6)	31.0 (16.9, 51.9)	34.4 (19.7, 55.9)	41.0
	Infl. A	253.5 (207.8, 306.3)	150.6 (116.5, 191.6)	187.9 (150.1, 232.3)	28.0 (14.9, 47.8)	155.0
	Infl. B	7.1 (1.5, 20.8)	182.5 (144.8, 227.1)	0.0 (0.0, 8.2)	0.0 (0.0, 7.9)	47.4
85 years or older	RSV	112.3 (36.5, 261.9)	386.5 (229.2, 610.2)	165.5 (71.5, 325.8)	214.8 (107.3, 384.0)	219.8
	Infl. A	1212.9 (912.5, 1579.7)	901.9 (650.7, 1217.1)	496.5 (318.4, 737.8)	58.6 (12.1, 171.1)	667.5
	Infl. B	44.9 (5.4, 162.2)	687.1 (470.5, 968.7)	0.0 (0.0, 76.3)	0.0 (0.0, 72.0)	183.0
Dominant influenza subtype/lineage based on national sentinel data		A(H3N2)^a^	A(H3N2)/B Yamagata	A(H3N2)/A(H1N1)pdm09	A(H1N1)pdm09	N/A
Vaccination coverage (65+‐year‐olds, study area)		56.4%	56.1%	57.9%	54.6%	56.3%

^a^ No detections of A(H1N1)pdm09.

Abbreviation: RSV, respiratory syncytial virus.

## 4 DISCUSSION

We observed a considerable and partially clinically undetected and underreported disease burden of laboratory‐confirmed RSV among hospitalized adults. In our prospective substudy, 6% of all SARI patients had RSV, and 20% had influenza. RSV diagnosis was more often missed on clinical grounds or omitted from medical records compared with influenza. Most patients in both groups had at least one preexisting comorbidity. However, based on McCabe scores, RSV patients had serious underlying comorbidity slightly more often than influenza patients (42% vs. 25%). The duration of hospitalization, need for ICU admission, or outcome did not differ between RSV and influenza patients, implying similar clinical severity. In our register‐based cohort substudy, the mean seasonal attack rates of RSV and influenza hospitalizations consistently increased with age and were 14 and 13 times higher among 65+ than 18‐ to 64‐year‐olds, respectively. Over four epidemic seasons, the mean attack rates of influenza were four to five times higher than those of RSV in all age groups. Attack rates of RSV and influenza hospitalizations varied remarkably between seasons.

Of the SARI patients included in our prospective substudy, 4% and 8% had RSV, and 25% and 14% had influenza during seasons 2018–2019 and 2019–2020, respectively. This is well‐aligned with findings from recent studies in which the proportions of RSV and influenza‐positive patients were 3–9% and 19–49%, respectively.^20^, ^22^, ^31^

During the 2019–2020 season, a nationwide lockdown due to the COVID‐19 pandemic began on March 17 (Week 12) and was followed by the disappearance of RSV and influenza cases in both substudies, while SARS‐CoV‐2 continued to circulate.^27^ In our retrospective cohort, this may partly be explained by a simultaneous decrease in testing for RSV and influenza. However, the majority of patients recruited to the prospective substudy continued to be tested for RSV and influenza, and no cases were found, which together with similar findings from other countries suggests that mitigation measures against SARS‐CoV‐2 are effective against RSV and influenza.^32^

In our retrospective cohort, no difference in the median ages of RSV, influenza A, and influenza B patients was observed when all four seasons 2016–2020 were included. This contrasts with many previous studies, which have found RSV patients to be older.^20^, ^21^, ^33^, ^34^ Interestingly, when only seasons 2018–2020 were considered, RSV patients were significantly older than influenza patients. During those seasons, the dominant influenza subtype in Finland was A(H1N1)pdm09, which seems to affect younger patients than A(H3N2), as also observed in our prospective substudy.^35^ Thus, the possible age difference between RSV and influenza patients may depend on the circulating influenza A subtype.

Consistently with previous research, the majority (30/31, 97%) of RSV patients had at least one preexisting comorbidity.^22^, ^23^, ^31^, ^33^, ^36^, ^37^ Serious comorbidities (McCabe 2 or 3) were somewhat more common (42% vs. 25%, *p* = 0.114) among RSV than influenza patients. As in previous studies, chronic kidney diseases were more common among RSV than influenza patients.^20^, ^21^ As in other studies, cancer and immunosuppression tended to be more common among RSV than influenza patients; however, these differences did not reach statistical significance, and the lack of patients with acute hematologic malignancies or recent transplant may have affected our results.^20^, ^21^, ^33^ Another recent study reported that hematologic malignancies and solid cancer are risk factors of RSV pneumonia.^38^

The clinical pictures of RSV and influenza were similar except that fever was less common among RSV patients as in previous studies.^5^, ^9^, ^31^, ^34^ Sudden onset of symptoms was less common, and hospitalization occurred on average 1 day later in RSV than influenza SARI. This later presentation and hospitalization of RSV patients have been documented in several studies.^11^, ^21^, ^22^, ^31^, ^33^ Consistently with other studies, the duration of hospitalization and need for ICU admission did not differ significantly between RSV and influenza patients.^6^, ^20^, ^21^, ^22^, ^33^

Notably, in our prospective substudy, 13% of RSV cases were missed based on clinical diagnostics alone, and the virus‐specific diagnosis was omitted from medical records by treating physicians in 48% of cases compared with 1% missed diagnoses and 15% of omitted diagnosis codes for influenza. Zhou et al found that while specific diagnosis for RSV was more often recorded among children, among 50–64 and 65+‐year‐olds, only 5.3% and 1.7% of estimated RSV‐associated and 17.3% and 14.7% of influenza‐associated hospitalizations listed specific diagnosis codes.^39^ Thus, considerable underestimation of the disease burden of RSV can occur in epidemiological studies relying on regular clinical practice and codification. In contrast, the virus‐specific diagnosis was present in the medical records of all our COVID‐19 positive prospective substudy patients (data not shown).

In our prospective substudy, none of either RSV or influenza patients died during hospitalization, whereas in other prospective studies, in‐hospital mortality has ranged from 0% to 8% for RSV and from 1% to 7% for influenza.^6^, ^20^, ^22^, ^36^ Furthermore, during follow‐up, none of either RSV or influenza patients died within 30 days, and only 2 (6%) of RSV and 1 (1%) of influenza patients died within 3 months from admission. In contrast, in a retrospective cohort study from Hong Kong, 30‐day and 60‐day all‐cause mortality of RSV and influenza patients were 9.1% and 8.0% and 11.9% and 8.8%, respectively.^31^ Also, two recent studies from China have found higher 30‐day mortality (10.9–13.7%) among RSV than influenza patients (5.0–6.2%).^21^, ^40^ Our low mortality rates are likely related to the underrepresentation of elderly patients as institutionalized patients, and those treated at secondary care hospital were excluded from our prospective substudy. Additionally, severely ill patients may have been unable to give their informed consent; however, we did try to overcome this limitation by accepting consent from their next of kin.

In our register‐based cohort study, among adults, attack rates of both RSV and influenza hospitalizations rose with age consistently with previous studies.^8^, ^39^, ^41^, ^42^ Altough most RSV hospitalizations occurred among the elderly, in our study as well as previous studies, work‐age adults with comorbidities were also at risk of being hospitalized with RSV.^6^, ^22^, ^41^ The mean seasonal attack rates of hospitalized influenza were almost fivefold compared with RSV both among 18‐ to 64‐year‐olds (20.1 vs. 4.1 per 100,000) and among 65+ elderly (264.6 vs. 58.3 per 100,000). The highest rates of RSV and influenza hospitalizations were seen among 85+ adults (mean 219.8 vs. 850.5 per 100,000) followed by 65‐ to 84‐year‐olds (41.0 vs. 202.4).

RSV and influenza‐associated hospitalizations have previously been estimated by several modeling studies giving varying results. In a U.S. study spanning 1993–2008, Zhou et al estimated similarly to our study fivefold mean hospitalization rates for influenza compared with RSV among 50‐ to 64‐year‐olds (65.6 vs. 12.8 per 100,000 person‐years) and almost fourfold (309.1 vs. 86.1) among 65+ elderly. Another U.S. modeling study by Matias et al spanning 1997–2009 reported higher annual mean rates of RSV‐related hospitalizations of 9, 28, 84, and 258 per 100,000 among 18–49, 50–64, 65–74, and 75+‐year‐olds, whereas the corresponding figures for influenza were 41, 117, 256, and 589.^39^, ^41^ In other modeling studies, mean seasonal RSV‐attributable hospitalizations among the elderly have ranged from 30 to 156 per 100,000.^8^, ^42^ Lower attack rates were reported by a U.S. active surveillance multicenter study, where estimated annual rates of RSV‐associated community‐acquired pneumonia hospitalizations were only 8/100,000 among 50‐ to 64‐year‐olds, 25/100,000 among 65‐ to 79‐year‐olds, and 50/100,000 among 80+‐year‐olds, possibly because they required radiologic evidence of pneumonia.^12^

Comparisons between studies are complicated by the inclusion of different populations and seasons, varying sensitivities and clinical use of laboratory tests, differences in viral surveillance strategies, differing practices regarding hospitalizations, and different statistical modeling methods used. Gilca et al obtained considerably varying estimates of virus‐attributable hospitalizations when six commonly used statistical methods utilizing the same viral surveillance data and hospital discharge database were compared with simultaneous prospective study data with laboratory‐confirmed RSV and influenza hospitalizations among children.^43^ They called for validation of statistical methods against prospective laboratory‐confirmed data. Although this validation is not in the scope of our study, our study offers a different perspective to assessing the population‐based attack rates of laboratory‐confirmed hospitalizations.

In our study, both RSV and influenza hospitalizations exhibited season‐to‐season variation, which was more pronounced for influenza probably related to dominating influenza subtype and seasonal IVE. Attack rates of RSV hospitalizations were highest in 2017–2018 followed by 2019–20, which is consistent with the biannual larger RSV epidemics observed in Finland.^15^, ^16^ As many as 0.09% and 0.3% of 65‐ to 84‐year‐olds and 0.4% and 1.6% of 85+ adult population were hospitalized with acute RSV or influenza during the harshest season 2017–2018. We observed more than tenfold variations of influenza hospitalizations by season, which for 2019–2020 may partially be explained by the effect of the COVID‐19 pandemic on the circulation of influenza, but variation was considerable also between other seasons. The highest influenza A attack rate among 65+ elderly (345 per 100,000) was observed in 2016–2017 when influenza A(H3N2) was the only influenza A virus subtype detected among sentinel samples in Finland. Similarly, attack rates of influenza‐associated hospitalizations have been highest for influenza A(H3N2) in previous studies.^39^, ^41^

We estimated 3148 (range 369–5399) influenza hospitalizations among the elderly in Finland each influenza season based on our attack rate calculations. In comparison, Jacks et al reported a yearly median of 322 (range 111–1176) influenza hospitalizations based on influenza‐specific discharge diagnoses among 65+‐year‐olds in Finland in 1996–2010.^44^ Our estimates of laboratory‐confirmed influenza hospitalizations among the elderly are almost tenfold compared with their figures. This discrepancy is likely explained by the continuous growth of the elderly population in Finland as well as the underdiagnosis of influenza during previous decades due to the lack of viral diagnostics and the use of less sensitive diagnostic tests.

Our findings highlight the need for more effective influenza vaccines and RSV vaccines especially among the elderly. In our retrospective cohort, attack rates of influenza among the elderly were high despite more than half of the 65+ elderly being vaccinated against influenza each season. Attack rates were especially high in A(H3N2)‐dominated influenza seasons, which is in line with the decreased IVE against A(H3N2).^45^, ^46^

Our prospective substudy setup offered several advantages. SARI patients were systematically screened and tested for both influenza and RSV by RT‐PCR, which is more sensitive than previously used viral detection methods. We included all 18+ adults, whereas many previous studies have focused on the elderly. This substudy also had limitations, most importantly its small sample size. All eligible patients did not consent to participate, and elderly patients were underrepresented. As our SARI definition did not include runny nose or wheezing commonly observed in RSV infection, RSV cases may have been missed.^5^, ^7^, ^11^

Strengths of our register‐based cohort substudy include utilizing data from four different epidemic seasons, which enabled the observation of the seasonal variation of these viruses, and comprehensive coverage of our background population. We believe our attack rates are accurate estimates of community‐acquired laboratory‐confirmed influenza hospitalizations given that the study site is the only regional reference center for the inhabitants needing hospital care and has the policy to test all hospitalized SARI cases with RT‐PCR, leading to the clinical detection of 99% of influenza cases in our prospective substudy.

Limitations of our retrospective cohort substudy include testing at clinician's discretion, likely leading to some missed diagnoses. Our attack rates of acute RSV‐associated hospitalizations are probably underestimates because based on our prospective substudy, 13% of RSV diagnoses were missed when only diagnostic clinical samples were considered and because patients with severe immunosuppression were treated at another hospital. Additionally, patients whose positive RSV or influenza test was taken before hospitalization at primary care or at a previous visit to JEC not leading to hospitalization are missing from attack rate calculations. However, RT‐PCR testing for influenza and RSV is uncommon at primary care. Our focus was on hospitalizations with acute laboratory‐confirmed RSV or influenza; thus, we did not include later hospitalizations due to, for example, secondary bacterial infections, exacerbations of preexisting conditions, or cardiorespiratory complications unless the patients were tested for and remained PCR‐positive for RSV or influenza, which becomes more unlikely the more time passes since the onset of infection.^47^ Thus, our attack rates should be interpreted as representative of the lower bounds of the true attack rates of RSV and influenza hospitalizations.

In conclusion, our study adds to the growing evidence indicating that besides influenza, RSV is an important and underestimated cause of hospitalizations for acute respiratory infection among the elderly. RSV vaccines and more effective influenza vaccines are needed to decrease this significant burden of severe respiratory infections in the elderly population and to alleviate the seasonally varying and thus unpredictable strain put on hospitals. A better understanding of RSV‐associated morbidity among adults is necessary to guide the use of preventive measures, diagnostics, and possible treatments of RSV.

## AUTHOR CONTRIBUTIONS

**Raija Auvinen:** Conceptualization; data curation; formal analysis; funding acquisition; investigation; methodology; project administration; resources; validation; visualization. **Ritva Syrjänen:** Conceptualization; formal analysis; funding acquisition; investigation; methodology; project administration; resources; supervision; validation; visualization. **Jukka Ollgren:** Formal analysis; methodology; software; supervision. **Hanna Nohynek:** Conceptualization; funding acquisition; methodology; project administration; resources; supervision. **Kirsi Skogberg:** Conceptualization; formal analysis; funding acquisition; methodology; project administration; resources; supervision.

## PATIENT CONSENT STATEMENT

Informed consent to participate was obtained from all participants of the prospective substudy. Informed consent included consent to publish study data of the prospective substudy.

## PERMISSION TO REPRODUCE MATERIAL FROM OTHER SOURCES

No material from other sources was reproduced.

### PEER REVIEW

The peer review history for this article is available at https://publons.com/publon/10.1111/irv.12914.

## Supporting information


**Table S1.** Characteristics of retrospective cohort patients by epidemic season and virus type.

## Data Availability

The data that support the findings of this study are available from the corresponding author upon reasonable request respecting GDPR and with permission from HUS and THL.
