# Supplementary material for: Clinical characteristics and population‐based attack rates of respiratory syncytial virus versus influenza hospitalizations among adults—An observational study
Source: Influenza Other Respir Viruses. 2021 Oct 3;16(2):276–88. doi: 10.1111/irv.12914 (PMC8818833; doi:10.1111/irv.12914)
Supplement: Supplementary file 1 — Table S1. Characteristics of retrospective cohort patients by epidemic season and virus type. [file IRV-16-276-s001.docx]

Supplement 1. Characteristics of retrospective cohort patients by epidemic season and virus type.

|  | **2016-17** |  | **2017-18** |  | **2018-19** |  | **2019-20** |  | **Total 2016-20** |  |
| --- | --- | --- | --- | --- | --- | --- | --- | --- | --- | --- |
|  | **N** | **%** | **N** | **%** | **N** | **%** | **N** | **%** | **N** | **%** |
| **Hospitalized patients at JEC, total** | 208 | 100 | 350 | 100 | 184 | 100 | 83 | 100 | 825 | 100 |
| **RSV** |  |  |  |  |  |  |  |  |  |  |
| **Total** | 15 | 100 | 69 | 100 | 26 | 100 | 39 | 100 | 149 | 100 |
| **Sex** |  |  |  |  |  |  |  |  |  |  |
| Women | 12 | 80 | 44 | 64 | 13 | 50 | 21 | 54 | 90 | 60 |
| Men | 3 | 20 | 25 | 36 | 13 | 50 | 18 | 46 | 59 | 40 |
| **Age** |  |  |  |  |  |  |  |  |  |  |
| Median (IQR)[range] | 68 (61,86)[46-95] |  | 78 (71,85)[22-97] |  | 80 (67,86)[36-90] |  | 75 (61,86)[24-97] |  | 77 (67,86)[22-97] |  |
| 18-64 years | 6 | 40 | 12 | 17 | 4 | 15 | 12 | 31 | 34 | 23 |
| 65-84 years | 4 | 27 | 39 | 57 | 14 | 54 | 16 | 41 | 73 | 49 |
| 85+ years | 5 | 33 | 18 | 26 | 8 | 31 | 11 | 28 | 42 | 28 |
| **Influenza A** |  |  |  |  |  |  |  |  |  |  |
| **Total** | 186 | 100 | 136 | 100 | 157 | 100 | 42 | 100 | 521 | 100 |
| **Sex** |  |  |  |  |  |  |  |  |  |  |
| Women | 103 | 55 | 71 | 52 | 88 | 56 | 15 | 36 | 277 | 53 |
| Men | 83 | 45 | 65 | 48 | 69 | 44 | 27 | 64 | 244 | 47 |
| **Age** |  |  |  |  |  |  |  |  |  |  |
| Median (IQR) [range] | 79 (71,85)[20-100] |  | 77(68,85)[28-97] |  | 74 (60,81)[18-96] |  | 56 (37,74)[19-90] |  | 76 (65,84)[18-100] |  |
| 18-64 years | 25 | 13 | 28 | 21 | 48 | 31 | 26 | 62 | 127 | 24 |
| 65-84 years | 107 | 58 | 66 | 49 | 85 | 54 | 13 | 31 | 271 | 52 |
| 85+ years | 54 | 29 | 42 | 31 | 24 | 15 | 3 | 7 | 123 | 24 |
| **Influenza B** |  |  |  |  |  |  |  |  |  |  |
| **Total** | 7 | 100 | 145 | 100 | 1 | 100 | 2 | 100 | 155 | 100 |
| **Sex** |  |  |  |  |  |  |  |  |  |  |
| Women | 3 | 43 | 92 | 63 | 0 | 0 | 2 | 100 | 97 | 63 |
| Men | 4 | 57 | 53 | 37 | 1 | 100 | 0 | 0 | 58 | 37 |
| **Age** |  |  |  |  |  |  |  |  |  |  |
| Median (IQR) [range] | 75 (57,85)[42-86] |  | 75 (65,84)[19-100] |  | 62 |  | 40 [37-42] |  | 75 (65,83)[19-100] |  |
| 18-64 years | 2 | 29 | 33 | 23 | 1 | 100 | 2 | 100 | 38 | 25 |
| 65-84 years | 3 | 43 | 80 | 55 | 0 | 0 | 0 | 0 | 83 | 54 |
| 85+ years | 2 | 29 | 32 | 22 | 0 | 0 | 0 | 0 | 34 | 22 |
